# Co-location as a Driver for Cross-Sectoral Collaboration with General Practitioners as Coordinators: The Case of a Danish Municipal Health Centre

**DOI:** 10.5334/ijic.2471

**Published:** 2016-12-05

**Authors:** Christian Elling Scheele, Karsten Vrangbæk

**Affiliations:** University of Copenhagen, Department of Public Health, Centre for Healthy Aging, Centre for Health Economics, DK

**Keywords:** Integrated care, co-location, cross-sectoral collaboration, municipal health centre

## Abstract

The issue of integrated care and inter-sectoral collaboration is on the health policy agenda in many countries. Yet, there is limited knowledge about the effects of the different policy instruments used to achieve this. This paper studies co-location as a driver for cross-sectoral collaboration with general practitioners (GPs) acting as coordinators in a municipal health centre. The purpose of the health centre, which is staffed by health professionals from municipal, regional and private sectors, is to provide primary health services to the citizens of the municipality. Co-locating these professionals is supposed to benefit e.g., elder citizens and patients with chronic diseases who frequently require services from health professionals across administrative sectors.

Methodologically, the analysis is based on qualitative data in the form of semi-structured interviews with the health professionals employed at the health centre and with administrative managers from municipal and regional government levels.

The study finds that co-location does not function as a driver for cross-sectoral collaboration in a health centre when GPs act as coordinators. Cross-sectoral collaboration is hampered by the general practitioners’ work routines and professional identity, by organisational factors and by a lack of clarity concerning the content of collaboration with regard to economic and professional incentives.

## Introduction

The municipal level of government in Denmark is responsible for health services in the primary care sector such as nursing homes, home care and home nursing, while the responsibility for rehabilitation services is shared between the regional and municipal levels. Primary sector health services paid for by the regional government level include, *inter alia*, self-employed GPs, chiropractors, chiropodists, otolaryngologist and rheumatologists, who are remunerated according to a fee-for-service scheme [1]. This shared responsibility between local and regional government causes coordination challenges [2].

The structure of health services in Denmark underwent significant changes in 2007 when a major administrative reform merged the previous 13 counties into five new regions. In addition, 98 new and larger municipalities replaced the previous 271 municipalities. At the same time, on-going reforms in the hospital sector have been and continue to drive the movement towards fewer, larger and more specialised hospitals. The increasing size of the municipalities opens up the possibility of new organisational models for primary sector health care provision [1]. For instance, new models have been called for because citizens in rural areas have to travel farther to receive hospital treatment, and because these areas are also experiencing difficulties attracting GPs [3].

After the 2007 reform, the concept of ‘municipal health centres’, which are located at a single address and host various health services provided by the local and regional governments as well as private health professionals, was introduced by Regional Denmark, the association of the five regions in Denmark [4]. The purpose of this initiative was to address the problems of sporadic access to medical care in rural Denmark and the cross-sectoral coordination challenges articulated within the context of ‘integrated care’ [Ibid.]. Recent epidemiological transitions and changing socio-demographics have also increased the relevance of integrated care within the context of a municipal health centre [5]. Denmark’s aging population has resulted in an increase in the number of patients with chronic diseases who need transversal primary sector health services [6,7]. The cost of the construction of health centres is in many cases shared by the local and regional governments in which the health centre is located. The national government has supported the concept of health centres by allocating subsidies in 2009–2010 and planned allocation again in 2015–2018 and by encouraging applicants to focus on cross-sectoral collaboration [8,9].

GPs have several roles as coordinators of health services. They act as gatekeepers that refer patients to secondary and tertiary healthcare services [1,3], and serve as the primary contact point for patients before and after hospital admission. As such, they play an important role of effective coordination according to the logics of integrated care [10,11,12,13]. According to the national agreement with Regional Denmark, GPs are obligated to contribute to increasing cross-sectoral collaboration locally [14]. This is especially relevant in connection with health centres, because GPs write referrals to other health professionals within the health centre (e.g., the psychologist or the chiropodist), and because treatment often occurs in collaboration between the GPs and the other professionals within the health centre. (For example, such collaboration occurs in the case of elderly multi-morbid patients who require wound care from municipal nurses and diabetes management by the GP.)

The health centre studied in this paper is organised as a ‘rent community’, whereby the municipality provides a common facility and the health professionals pay for their own offices and their share of the costs for waiting areas, kitchen facilities, cleaning services, etc. [15]. The health centre was opened ultimo 2013 and fully leased by the end of 2014, and focuses on three groups of patients: citizens with chronic diseases, the elderly and the mentally ill [15]. This form of cross-sectoral collaboration aimed at clearly defined groups of patients can be understood as a micro-level clinical integration of care [16,17]. There is no overarching management structure for municipal, regional and private health professionals. Rather than relying on management structures, it is assumed that co-location of the health professionals will promote cross sectoral collaborations [11]. The primary argument for a shared location (which also appears in the project description for the health centre [15]) is that the combining of multiple health professions in one place will enhance information transaction, facilitate communication and increase personal familiarity between the health professionals [18 p. 143]. Yet, some scholars have raised concerns that attempts to promote cross-sectoral coordination through co-location have the potential to generate clashes between different individuals, professions and cultures that create barriers to collaboration [11,19]. This leads to the following research question for this paper: *‘Does co-location of health professionals function as a driver for increased cross-sectoral collaboration with general practitioners as coordinators?’*

## Theory and methods

### Theory

In this paper, integrated care refers to a coordinated form of cooperation where each actor’s activities are clearly defined and where a mutual knowledge of working methods, procedures and conditions is established. In this sense, ‘integration’ can be understood as an endpoint of the coordination process [5,20]. Accordingly, ‘integrated care’ is used as an ideal type concept [21] that covers those forms of collaboration that narrow inter-sectoral gaps [22]. This means that the term signifies a broader understanding of care, rather than very specific patient flows.

To date, ‘Co-location as driver for cross-sectoral collaboration’ has not been examined theoretically within the context of integrated care. At the centre of the concept of *co-location* stands *spatial proximity* [23]. Therefore, this study can be theoretically conceived of as a case of *spatial proximity as driver collaboration*. Relevant literature that addresses spatial proximity as a driver for collaboration can be found within the realm of economic and scientific collaborations [24,25,26,27]. First, proximity has been found to drive the quality and quantity of communication, as it takes relative little effort for actors to interact with each other, which simultaneously promotes collaboration and increases trust [24 p. 140, 27]. Second, proximity increases the frequency of communication among both potential and existing work partners [28,29]. Third, the likelihood of chance encounters among people who have the same prerequisites for communication increase because proximity allows actors to pick up information opportunistically about a co-located actor’s availability [30]. However, proximity can also lead to interruptions and loss of privacy in situations where planned communication is more desirable [31].

Considering that this study was conducted approximately one year after the health centre was fully leased, it is relevant to consider the barriers that can be found within implementation theory [32]. These include the lack of adequate time and sufficient resources, poor understanding of, or disagreement about, objectives, tasks not being fully specified in the correct sequence, lack of trust-building activities, and lack of effective leadership [33]. A literature review of cross-sectoral collaboration identifies similar barriers and generally concludes *‘that the normal expectation ought to be that success will be very difficult to achieve in cross-sectoral collaborations’* [34 p. 52].

Co-location is generally recognised as a potential driver for integrated care, although a review of papers concludes that the empirical evidence is mixed [35]. Some studies point to the fact that co-location is a necessary prerequisite for integrated care [36,37,38]. A recent study found that co-location of multiple disciplines within a primary care practice was associated with increased capacity to provide broad, specialised and preventive care for people with chronic disease [39 p. 5]. More specifically, studies demonstrate that there are positive associations between the number of co-located disciplines and the amount of consultations involving older adults, patients with diabetes or hypertension, and increased participation in disease management programs for diabetes, COPD and asthma, among other diseases [Ibid.]. Furthermore, co-location seems particularly effective when combining psychiatric health services with primary care [40,41,42]. However, other studies find that co-location is not a key factor for cross-sectoral collaboration [43,44]. Previous empirical research has also pointed out that factors other than distance may function as barriers for cross-sectoral collaboration, including differences in funding mechanisms, organisational settings and working cultures [19,45,46,47,48].

Theoretically, there is an argument for co-location understood as proximity as a driver for collaboration. However, this relationship is under theorised within the context of primary health care cross-sectoral collaboration. The empirical evidence of co-location as a driver in connection with integration of care is mixed. The expectations of cross-sectoral collaborations’ success in general ought to be low, and the expectations of co-location as a driver for cross-sectoral collaboration in this case are medium to low.

### Case-selection

Due to the low expectations of co-location as a driver for inter-sectoral collaboration, the case selection for this paper’s case study pursued a ‘most likely’ principle strategy. The purpose was to create the basis for a critical case in the sense that if co-location does not drive cross-sectoral collaboration in this instance, it will not likely occur in other cases [49]. At the time of the case selection process, there were 25 health centres in Denmark [50]. The first step of the case selection process consisted of presenting the research project to administrative division managers in the five regional governments. This was necessary, because regional government officials would have to allow interviews of regional government administrative staff, and because they could assist in identifying a ‘most likely’ case. One regional government expressed interest in the study and suggested a number of relevant health centres.

This process was combined with the study of the project descriptions underlying all of the regions’ health centres in order to identify those that specifically emphasised co-location as a strategy to obtain cross-sectoral collaboration. Based on this process, two equally relevant cases were identified [15,51]. However, only one of these municipalities agreed to participate. The health professionals working at that health centre were invited to a meeting where the research project was presented in order to secure their support for the project.

The original idea behind the selected health centre came from three GPs who were interested in sharing the same address in the municipality. The health centre was financed jointly by the municipality and the region along with an initial grant of approximately 9.9 million DKK from the national government’s Ministry of Health and Disease Prevention. Private practicing health professionals approved by Danish Regions were able to rent offices within the health centre on a ‘first come – first served’ basis as selection criteria. The present configuration of the health centre is illustrated in Table 1 below:

**Table 1:** Municipal health centre configuration.

Local Government Sector	Regional Government Sector	Private practicing sector

Physiotherapy	4 GPs + staff	Paediatric psychologist
Home care	Midwife	Chiropodist
Home nursing		Chiropractor
Physical rehabilitation		Paediatric occupational
Health clinic (disease prevention + health promotion)		therapy Health coach Reflexive therapy

The project description for the health centre, which was a key element in the grant application, emphasised ‘co-location’ as the primary driver for cross-sectoral collaboration. The project description argues that the health centre enables *‘cooperation across professions due to the [common] physical facilities… in such a way that there is an organisational breeding ground for an extensive collaboration across [municipal, regional and private] sectors…. The idea is that collaboration between GPs and [municipal] home care is developed around the elderly and patients with chronic disease or mental illness. [Furthermore, the health centre will provide] improved possibilities for round table discussions with representation by GPs and the municipality’* [15 p. 4]. In addition, the health centre is intended to improve collaboration because *‘barriers that exist in relation to cooperation across professions and [administrative] sectors are expected to be broken down because professionals are in close physical proximity to each other’* [Ibid.]. The health centre will enable *‘entering into local agreements [between the municipal actors and] the GPs that can facilitate collaboration that the labour market agreements do not create the room for’* [Ibid. p. 5]. The project description points out that cross-sectoral treatment is particularly relevant for the *‘chronic care patients, as they require health services from e.g., home care, disease prevention units, health promotion and frequent visits to their GP, while others need a coordinated effort from health and social services’* [Ibid.].

The expectation of the GPs’ role as coordinators of cross-sectoral collaboration is not only visible in the project description for the health centre. Two other strategic documents developed by the regional and local governments address the issue. First, the ‘Health Plan’ [46] for the regional government states: *‘It is a target [within the health centre] to develop patient pathways in a close cross-sectoral collaboration between the actors in the [health] centre. Accordingly, the health centre is to function as something else and more than a rental community… in order to provide health services as integrated care’* [52 p. 39]. Second, the so called ‘Plan for General Practitioners’, which contains the regional government’s plan for implementing their health care agreement with the GPs, states that: ’*Within the health centres health services are developed that provide coherence for the patients, more effective patient pathways, co-location of multiple professions and thereby a higher treatment quality and an expanded capacity among other because the possibilities for cross-professional and cross-sectoral coordination, sparring and collaboration are expanded’* [53 p. 20].

### Method

This study is based on qualitative data from 20 semi-structured interviews carried out from 12 December 2015 to 23 March 2016. Table 2 contains titles of the respondents organized according to sector. The respondents from the regional government were identified when contacting the administrative division managers in connection with the case-selection process. They pointed to the CEO of Department of Social Services and Labour, who listed municipal administrative relevant for the project and provided an oversight of the tenants in the health centre. The GPs were selected based on the research question, while the rest of the health professionals were identified based on a screening process conducted by telephone involving the health professionals and the municipal administrative staff. In this process, the actors were asked to identify health professionals most likely to have an incentive to enter into cross-sectoral collaborations with GPs as coordinators. The respondents signed a consent form committing them to confidentiality before the interviews. The interviews were carried out, recorded, and transcribed by the author with assistance from a public health master student with experience as an interviewer. Key themes from the interview guides are depicted in Table 3.

**Table 2:** Respondents

Sectors	Administrators (interview)	Health professionals (interview)

Regional Government	Head of Division (a) Head of Section (b)	GP1 (j), GP2 (k) GP3 (l), GP4 (m)
Local Government	CEO of Department of Social Services and Labour Market (c Executive officer from Administration of Social Services and Labour Market (d) Head of Division of Labour Market (e) Head of Division of Active Nursing and Care (f) Head of Division of Health and Rehabilitation (g) Head of Nursing (h) Head of Rehabilitation (i)	Physiotherapist 1 (n) Physiotherapist 2 (o) Nurse 1 (p) Nurse 2 (q)
Private sector	N.a.	Psychologist (r) Chiropodist (s) Chiropractor (t)

**Table 3:** Key themes from the interview guides.

Respondents	

All	Respondent’s background and role concerning the health centre; perceptions of communication between actors in the establishment phase/operation phase; respondents’ function in and expectations of the health centre; benefits for citizens/patients, understanding of the implications of ‘co-location’; suggestions of forms of cross-sectoral collaborations; expectations of/experiences with GPs as coordinators within health centre; relationship between administrators and health professionals
Local and regional administrators	Motivation for establishing centre; strategic/everyday management of health centre; strategic agreements’ impact on cross-sectoral collaboration; configuration of health centre; initiatives taken to increase cross-sectoral collaboration with GPs as coordinators and possible examples hereof
Health professionals within the health centre	Motivation for moving into health centre; description of a normal workday; types of patients; benefits of co-location experienced personally/disadvantages of being located within the health centre; relationships between health professionals; possible patient benefits from cross-sectoral collaborations; barriers/drivers

The following table illustrates key themes from the interview guides:

The analysis followed the steps of Attride-Stirling’s thematic network analysis, which in this case entails a hybrid process of inductive and deductive coding [54]. The methodological approach integrates data-driven codes with theory-driven codes based on the reviewed literature [55]. Step 1 consisted of coding text segments with an interpretative title. This process generated a total of 46 codes. How the codes interconnected was then inductively explored, which resulted in the identification of 13 basic themes, cf. Step 2 in the thematic network analysis approach [54].

These basic themes highlight the factors that influence co-location as a driver for cross-sectoral collaboration as perceived by the interviewees. Based on the reviewed literature, these factors were deductively clustered into higher-order themes called ‘organising themes’ (Step 3). This process gave rise to the dimensions in the thematic network analysis. These were deductively (based on the paper’s research question) assembled into the analysis’ global theme of ‘co-location as a driver for cross-sectoral collaboration with GPs as coordinators in the health centre’ (Step 4). This process is illustrated in Table 4, which outlines the different steps of the analysis.

**Table 4:** Global theme: Co-location as driver for cross-sectoral collaboration.

Thematic network analysis [54]

Basic themes: Factors influencing co-location as a driver for cross-sectoral collaboration with GPs as coordinators (interviews containing the code cf. Table 2)	Organizing themes: Dimensions	Global theme

Co-location facilitates – non-planned cross-sectoral communication (b, c, f, i, j, k, l, m, o, p, r)	Personal relations, trust and communication as drivers	Co-location as driver for cross-sectoral collaboration with GPs as coordinators in health centre
Increased communication – raise awareness of the identity of other actors thereby developing relationships (a, h, i, j, m, p, q)		
GPs’ work schedules and treatment approaches impede cross-sectoral collaboration generated by co-location (a, g, j, k, l, n, o, p, q)	GPs’ work routines and professional identity as barriers	
GPs perceive that they work in a ‘Doctors Clinic’ instead of a ‘Health Centre’ (a, j, k, l, m, t)		
GPs not interested in social activities or general meetings with co-located health professionals (b, j, k, m, r, s, t)		
GPs’ collective agreement undermines implementation of strategic agreements (‘Health Agreement’ and ‘Plan for GP’) that commits GPs to be cross-sectoral coordinators in health centre (a, b, j, k, l, m)	Unaligned economic incentives as a barrier	
GPs activity-based remuneration and municipal health professionals’ monthly salaries impede cross-sectoral collaboration (a, b, c, f, g, j, k, l, m, r)		
Lack of evidence/suggestions concerning cross-sectoral collaboration with GPs as coordinators (a, c, e, h, i, j, l, r)	Lack of clarity concerning the content of collaboration as a barrier	
Lack of clarity concerning the location of responsibility for developing content of cross-sectoral collaboration (a, b, c, j, k, m, n, o, t)		
Health centre effectively functions as rental co-op (no admission criteria for private health professionals other than willingness to pay rent) (b, c, f, g, r, t)	Organisational issues as barriers	
Cross-sectoral collaboration driven by co-location primarily included in initial project description in order to obtain national funding (d, l)		
Both local and regional government administrative levels and health professionals within the health centre are passive and fail explicitly to request cross-sectoral collaborations involving GPs (a, c, e, f, g, h, i, j, l, m, n, p, r, s, t)		
Lack of common vision/goals/organisation concerning cross-sectoral collaboration with GPs as coordinators in the health centre (a, b, c, f, g, l, n, r, t)		

Table 4 is used to structure the presentation of results, where quotes are included to illustrate nuances and connections in the material, and to structure the discussion.

## Results

### Personal relations, trust and communication as drivers for cross-sectoral collaboration

The only example of cross-sectoral collaboration that did not exist before the creation of the health centre occurs in the intersection of the GPs and the municipal home nursing and rehabilitation units. As the GPs get to know the municipal home nurses personally, communication and trust improves and the sectoral gap narrows. One GP [GP1] said: *‘…it is really nice that I have gotten to know “Bridget” [municipal health professional] that I call on the phone. The tone changes. I know that when Bridget calls there is an important problem that we collectively need to address. We obtain a closer collaboration and better understanding of each other and the patients [that we share]’*. This experience is shared by the home nurse [N1]: *‘I think that [the benefit of co-location] is that it much easier to grab hold of a GP. In our [municipal] health care unit, elderly citizens receive wound care… blood sugar controls or blood pressure measurements… and if the citizen is in a really bad state or if the wound has severely changed, we can grab hold of a doctor and say “Could you come over right away and look at this wound!” … the citizen experiences coherence… that we are multiple collaborators in the same house’*. Even though these statements only demonstrate a weak form of the integration of informal ad hoc collaboration, they illustrate that co-location does create a stronger basis for collaboration.

### GPs’ work routines and professional identity as barriers

The GPs perceive that their work approach and busy workday with back-to-back patient consultations inhibits cross-sectoral communication. When asked about communication with the other actors in the health centre, one GP [GP2] replied: *‘It does not exceed what we otherwise would have if we had our own [doctors’ clinic]… That’s ok, because our workdays are highly different and we have different approaches to our work [compared to the other health professionals].’…’Our job is to treat patients – not to have meetings [with actors from the other sectors discussing collaborations]’*. The GPs perceive that they are different from other health professionals [GP2]: *‘So, all of those activities concerning social issues and get-togethers and spending time on meetings is not something that doctors prioritise. And I think that the other personal groups would like to hold meetings and use up much of our time’*.

The quote above illustrates indirectly that the GPs do not feel part of the health centre. They even disclose this more directly. [GP3]: *‘I conceive of [the health centre] as a doctors’ clinic… It was the municipal administration that came with the ‘health centre agenda’. [The health centre model] was not a part of [our original agenda]. Absolutely not!’* This quote also indicates that there is a misalignment between the GPs’ understanding of the health centre and the goals in the project description as formulated by the municipality.

The GPs neither request nor perceive that co-location with health professionals from other sectors create new benefits: [GP1]: *‘Well, we [the GPs] do not really have a great need for collaborating with the other [sector’s] actors’*. [GP3]: *‘… Well, if you get down to brass tacks, I find it difficult to argue that working in a health centre has a great advantage. Our everyday works in the exact same way as if [we GPs] had been located by ourselves somewhere else in town’*.

Other health professionals also conceive of the GPs’ work flow as a barrier. [Physiotherapist 1]: *‘The challenge [concerning collaboration with the GPs] is probably the doctors’ work flow…. that they have a patient like every tenth minute’*. At the regional executive administrative level, there is an understanding of GPs’ professional culture that mirrors somewhat how GPs understand themselves, namely as professionals who are not particularly concerned with, nor incentivised to focus on cross-sectoral collaboration. [Regional Government Head of Division]: *‘[The GP’s] obtain a mono-professional education. They have a mono-professional approach to their work. “It is me and my patient”. They have mono-professional incentives. All our agreements, the way in which we remunerate and everything we tell them focus their attention on the individual patient [rather than on cross-sectoral collaboration]. All other things surrounding them are not that important’*. The issue of remuneration structures overlaps the next section, which concerns unaligned economic incentives as a barrier.

### Unaligned economic incentives as a barrier

GPs in Denmark are paid a combination of per capita and fee-for-service schemes. This reduces their incentive to allocate time to activities that are not directly covered by activity remuneration or is associated with a low remuneration fee. Accordingly, GPs have only a minor incentive to allocate time to discuss, develop and implement cross-sectoral collaborations, because these activities take time away from those that are more lucrative. This is in contrast to municipally employed health professionals who receive a fixed monthly salary. The GPs experience this as a barrier that curbs co-location as a driver for cross-sectoral collaboration: [GP1]: *‘That is exactly the core of the problem in making cross-sectoral collaboration function, because I do not think that the [municipal health professionals] understand our working conditions. It is very easy to take a lot of time out of your calendar …for cross-sectoral collaboration when your salary is fixed. However, we [GPs] earn money according to the amount of patients that we see in our consultation… we cannot afford to take two hours out of our schedule to [focus on collaborations] without payment’*.

As mentioned, there are two strategic governance agreements – ‘Health Plan’ and ‘Plan for General Practitioners’ – that specifically emphasise the GPs’ responsibility as cross-sectoral coordinators in the health centre structure. When confronted with these documents, one GP [GP3] responded: *‘It [the text in the agreements] is something that you would say in a toast at a festive arrangement [i.e., something to make all listeners happy]. Ha,ha… It sounds really good, but it does not fit within our everyday life’*. Simply put, the general GP explanation for not complying with these agreements is related to the incompatibility of their remuneration structures and work schedules vis-à-vis other actors’ remuneration and work schedules. One GP [GP3] said: *‘What really, really separates us from the [municipal health professionals] is that we are pressured for time. Even though they [the municipal actors] want to have meetings with us [to discuss cross-sectoral collaborations], we cannot close down our business…. They [the municipality] do not want to pay us for our time and they do not understand that we have a business to run’*. [GP1]: *‘I am surprised that our employee organisation has reviewed the agreement [Plan for General Practitioners]. They look good, but our remuneration agreement [national contract] is incompatible with us [being cross-sectoral coordinators within the health centre].’*

### Lack of clarity concerning the content of collaboration as a barrier

Most of the respondents support implementation of cross-sectoral collaboration in the health centre. However, no one is able to specify exactly the content of such collaborations: [Interviewer]: *‘… I have spoken with the municipality, and they tell me that they would like more [cross-sectoral] collaboration [in the health centre]. And then I ask them “Collaboration about what?”’* [GP3]: *‘Yes, about what? I don’t know that either. I don’t know what they imagine that we should collaborate more on. They have never been specific about [the content of the cross-sectoral collaborations]’*. When presenting a list of potential cross-sectoral collaborations provided by the local and regional government, the GPs comment that those collaborations have always existed. [GP3]: *‘We are already carrying out [all those forms of collaborations].’* [GP1]: *‘There is nothing new in those forms of collaboration. We are already carrying them out’*.

The Chief Executive Officer (CEO) of the municipal Department of Social Services and Labour Market believes that GPs are responsible for providing the descriptions of the content of the cross-sectoral collaboration: *‘The GPs have to tell me that [the content of the collaborations]… otherwise we don’t have a health centre where we collaborate. Then we only have rent co-op with a common address. And that was not the purpose’*.

The challenge of developing context-specific cross-sectoral collaborations with GPs as coordinators is considerable. The head of the municipal Division of Health and Rehabilitation said: *‘What I spend a lot of time pondering on is identifying [content for cross-sectoral collaborations] with the right incentives for the GPs – otherwise we cannot get them on board. The GPs’ work has to become easier… I cannot make their work harder if they are going to be part of [future cross-sectoral collaborations]. It has to be such that the GPs perceive that they get a good experience from working with the municipal or private actors. Then I can get the GPs to collaborate with me, but that is the nut I have to crack…’.*

When the municipal CEO of the Department of Social Services and Labour Market was asked what she had done to develop new forms of collaboration, she admitted that the municipality had failed. She [CEO] said: *‘No, we have not had those talks.’… ‘We have not done things any differently in the health centre compared to our other municipal health clinics [which only have municipally employed staff that focus on disease prevention] . …that I unfortunately have to admit’*.

### Organisational issues as barriers

Organisational factors that affect co-location as a driver for cross-sectoral collaboration concern e.g., the configuration of actors within the health centre, common vision and shared goals.

When the municipality had to decide which health professionals to include in the health centre, the primary selection criteria was the willingness to pay rent: [Head of Division of Health and Rehabilitation]: *‘[The municipality’s] aim with the health centre was to create [an environment] with life… we haven’t focused on what the [health professionals from different sectors] could do for each other… there has not been strategic thinking from the municipality’s side other than we need as many actors as possible…’*. Another executive administrator from social services was even more direct when he concluded that cross-sectoral collaborations in the health care centre do not exist now and never will, because of the lack of strategic thinking in connection with the configuration of actors within the health centre.

Including cross-sectoral collaboration in the project description was done to increase the chances of obtaining funding from the national government. An executive administrator from social services administration states: *‘…we read [the material from the Ministry of Health and Prevention that emphasised cross-sectoral collaboration] in order to receive money…* (he whistles)*… we are not stupid. Then you include it in the project description without thinking about the consequences. We did not consider the professional nor economic outcomes that [co-location of health professionals] should result in’*.

None of the GPs reported that staff from the municipal or regional government contacted them and requested that they develop or implement cross-sectoral collaborations within the health centre. According to the GPs, the private health professionals did not request collaboration with them. When the chiropodists did communicate with the GPs within the context of the health centre, their communication did not go beyond the type of discussions that occurred before the creation of the health centre. In the case of the paediatric psychologist practising privately, the GPs referred patients to her independently of her being located in the health centre. She described cross-sectoral collaboration as *‘a possibility, but not someone that is substantial’*. In general, the private health professionals’ demand for cross-sectoral collaboration is vague, as expressed by a physiotherapist when asked whether she is interested in cross-sectoral collaboration with the GPs [Physiotherapist 1]: *‘Yes… we just don’t quite know to what extent we could be interested in [cross-sectoral collaboration with the GPs]’*.

One of the municipal respondents did not see any potential of cross-sectoral collaboration within the health centre: [The Head of Division of Labour Market]: *‘I simply do not in any way have a clear idea of how [cross-sectoral collaboration] could be developed in order to support the employment efforts…’*.

None of the interviews indicate that there are any forms of dialogue within the health centre concerning cross-sectoral collaborations. This may be explained by the lack of a relevant common vision: [Regional Government Head of Division]: *‘We are miles from something that could be imagined as common aims concerning the [cross-sectoral] functioning of the health centre’*. Even though there was one attempt to conduct a seminar to discuss physical and organisational parameters for cross-sectoral collaboration, it was not successful according to the GPs. [GP3]: *‘In the upstart period of the health centre, there was a workshop [aimed at discussing physical and organizational parameters for cross-sectoral collaboration] with actors from all sectors within the health centre. It was a waste of time. I was really grumpy when I left’*.

The lack of focus on organisational factors is shared by municipal health professionals: [Head of Rehabilitation]: *‘There has not been a focus on overarching issues [concerning cross-sectoral collaboration]… how to say it… the organisational issues are dragging behind because the attention has been on what our rooms should look like’*. These quotes demonstrate that organisational issues related to cross-sectoral collaboration have not been addressed within the health centre.

The majority of the barriers to cross-sectoral collaboration relate to the GPs and the administrative levels. It was within these interviews that the most informative and nuanced statements were made. However, codes from the interviews with the private health professionals support the findings demonstrated in Table 4.

## Discussion

There were few examples of co-location improving collaboration by facilitating informal, ad hoc cross-sectoral interaction between municipal home nurses and the GPs. This confirms that proximity increases the likelihood of chance encounters among people who have the same prerequisites for communication, because it allowed the nurses to pick up information opportunistically about GPs’ availability to them [30]. However, the majority of findings document barriers: the GPs’ primary focus on their doctor-patient relationship is institutionally reinforced by their national remuneration contract, which incentivises back-to-back patient consultations over e.g., cross-sectoral collaboration, which indicates a lack of adequate time and resources as well as competing institutional logics [3 p. 36, 33, 34]. The GPs who were interviewed reflected this notion of primarily orienting inward when they said they perceive that they work in a ‘Doctors House’ – not a ‘Health Centre’. Moreover, they said they are not interested in either professional or social meetings with the other health professionals within the health centre. The GPs’ behaviour reinforces the problem: it has been demonstrated that cross-sectoral collaboration requires time to develop trusting relationships. The lack of access to GPs, the GPs’ reluctance to collaborate and professional isolation impede cross-sectoral collaboration [47,48,56,57].

The interviewed GPs emphasised that they run a business; they do not perceive an economic incentive that is sufficient enough to address cross-sectoral collaboration – all of the GPs declared that they are always fully booked. However, at the same time, the GPs also said that they would have available time if the municipality would compensate them financially, which indicates that statements about needing the time for patients (i.e., a professional explanation) are also financially rooted. Accordingly, it may ultimately be necessary to change the national GP remuneration contract in order to secure the desired economic incentives, which illustrates the importance of the availability of time and economic resources [33,58].

Evidence-based knowledge concerning relevant types of collaborations within the health centre does not exist. This is a known barrier in connection with the political ambitions of implementing integrated care [19]. A relevant type of collaboration could be roundtable discussions with health professionals within the health centre that focus on individual patients. Such discussions could also include municipal social workers or unemployment consultants in order to help citizens suffering from somatic and social issues. An alternative could be seminars to develop coordinated cross-sectoral treatment programmes at the health centre that would target e.g., the multi-morbid elderly or patients suffering from one or multiple non- communicable diseases*.* The inability to decide upon the content of such collaborations within the health centre indicates a lack of leadership and a poor understanding of, and disagreement about, the objectives that could lead to cross-sectoral collaborations – which result in their not being developed [33,34]. The municipal CEO expects that the GPs take responsibility for developing the content of the collaborations. However, at the same time, she admits that she failed to discuss the responsibility of developing collaborations with the GPs after the health centre began to operate, which demonstrates a lack of leadership that results in collaboration tasks not being specified in the correct sequence [Ibid.].

The executive officer that said including the formulations of implementing cross-sectoral collaboration in the project description in order to increase the likelihood of receiving the state grant was done without *‘thinking about the consequences’*. This lack of considering the consequences is also reflected in the selection criteria for inclusion of health professionals, i.e., a willingness to pay rent and *‘creating of an environment with life’*, rather than a commitment to future tenants collaborating across sectors. This demonstrates a lack of leadership [Ibid.].

Following their receiving the necessary funds to create the health centre, the municipal administration should have developed a shared vision for the health centre actors. This is a critical step in establishing organisational configurations, because the dependency relations between co-location and cross-sectoral collaboration are multiple, as described above [15,33,59 p. 6]. Second, the vision seminar that was a failure in the eyes of the GPs should have been followed up by networking activities that are central when implementing coordinated care in new organisations [60]. In addition, activities aimed at improving respect, trust [34] and communication should have been carried out, which is important to the securing of a thorough and shared understanding of objectives [33] *in casu* cross-sectoral collaboration in primary health care [61,62].

### Limitations

Because the availability of health centres to this study was a factor in the case selection, there is the risk that other health centres that are organised differently might have been more appropriate as a ‘most likely case’. However, this is not very likely, as health centres are required to implement the same fundamental organisational design in order to obtain national funding [63]. Nevertheless, because of the parameters of the selection process, this study aims at analytical generalisability that relates the findings to the theory of co-location – understood as proximity – as a driver for collaboration [64].

## Conclusion

The purpose of this study was to find out if co-location of health professionals functions as a driver for increased cross-sectoral collaboration with GPs as coordinators in the case of a Danish health centre. Based on the findings, co-location does not qualify as a driver for cross-sectoral collaboration on its own within the context of a health centre.

In this case, cross-sectoral collaboration failed extensively because of barriers that can be traced back to a lack of leadership. The health centre selection criteria defined by the municipality did not obligate health professionals to engage constructively in cross-sectoral collaborations. No one took responsibility for developing the organisation within the health centre, or for the development of cross-sectoral collaborations that included professional and economic incentives for all participants. Co-location as a driver for cross-sectoral collaboration is an insufficient approach to a challenge that requires leadership that aligns initial conditions, attitudes, organisations and processes, and addresses interdependencies, while securing outcome and compliance [34].
